# The role of specialized outpatient palliative care in emergency advance directives: fewer hospitalizations, greater alignment with patient wishes

**DOI:** 10.1186/s13049-025-01468-2

**Published:** 2025-09-12

**Authors:** Yann-Nicolas Batzler, Jannis Wetzlar, Dirk Wildner, Manuela Schallenburger, Theresa Tenge, Marc Stefaniak, Miguel Angel Méndez Delgado, Jacqueline Schwartz, Lennert Böhm, Michael Bernhard, Martin Neukirchen

**Affiliations:** 1 Centre of Palliative Care and Pediatric Pain, Faculty of Medicine, Saarland University Medical Center and Saarland University, Homburg/Saar, Germany; 2 Interdisciplinary Center for Palliative Medicine, Medical Faculty and University Hospital Düsseldorf, Heinrich-Heine-University Düsseldorf, Moorenstraße 5, 40225 Düsseldorf, Germany; 3 Centrum für Integrierte Onkologie Aachen Bonn Cologne Düsseldorf (CIO ABCD), Aachen, Bonn, Köln, Düsseldorf, Deutschland; 4 Department of Anesthesiology, Medical Faculty, University Hospital Düsseldorf, Heinrich-Heine-University Düsseldorf, Düsseldorf, Germany; 5 Emergency Department, Medical Faculty, University Hospital Düsseldorf, Heinrich-Heine-University Düsseldorf, Düsseldorf, Germany

**Keywords:** Palliative care, Home care, Advance directives, Health care providers, Specialized outpatient palliative care, Palliative emergency care

## Abstract

**Context:**

Specialized outpatient palliative care (SOPC) aims at relieving symptoms and providing psychosocial aid in the outpatient setting. SOPC also supports patients in setting up advance directives so that their will is respected in emergency situations.

**Objectives:**

The aim of this study is to analyze the impact of SOPC on the completion of advance care directives with the focus on medical emergency identification (ID) cards (= living will in a credit card format on yellow paper) and their impact on hospital admissions.

**Methods:**

All adult patients who were admitted to the SOPC service at a tertiary care center in Germany between 07/2022 and 06/2023 were included in this retrospective cohort study. Demographic data, level of care, information on advance care directives, hospitalizations, adherence to patient wishes, and tumor specific treatments were collected. The data were analyzed using descriptive and inferential measures.

**Results:**

During the study period, 359 patients were included (52.0% female, mean age 74 ± 13 years). A medical emergency ID card was set up by 32.6% (*n* = 117/359). It was significantly more likely to be created during SOPC than prior to SOPC involvement (before: 20%, after: 80%; *p* < 0.05). Patients who expressed not wanting to be admitted to hospital in their medical emergency ID card saw significantly less hospital admissions (*p* < 0.05).

**Conclusion:**

SOPC supports patients in setting up medical emergency ID cards. These help in respecting patients’ wishes and prevent unwanted admissions to hospitals, thereby reducing strain on emergency services and emergency departments.

## Background

Advances in modern medicine have significantly increased life expectancy over recent decades. As a result, the proportion of older individuals within the population continues to rise, leading to a growing prevalence of chronic, non-communicable, and often incurable diseases. Many patients affected by these conditions suffer from a substantial burden of distressing symptoms, particularly in the final stages of life.

Acute or unexpected symptom crises may lead to an activation of emergency medicine services (EMS) with subsequent presentation to emergency departments (ED) and initiation of treatments that may not align with patients’ wishes [1]. In Germany, patients with palliative care needs account for approximately 10% of all EMS deployments [2] and a similar proportion of ED presentations [3]. However, with their primary focus on life-saving, both EMS services and EDs are often ill-equipped to address the specific needs of this population.

In the absence of decision-making capacity, advance directives ensure that patients’ wishes are respected. In Germany, these include the “*Vorsorgevollmacht*” (lasting power of attorney), which authorizes a designated individual to make healthcare decisions on the patient’s behalf and the “*Patientenverfügung*” (living will), which outlines the patient’s wishes regarding medical treatment in a legally binding way. However, in emergency situations, they are often inaccessible or difficult to interpret [4], which may lead to overly aggressive care based on the principle of “in dubio pro vita” (when in doubt, prioritize life).

To address this issue and support faster recognition of patient preferences, in 2018 the Düsseldorf Medical Emergency ID Card was developed in collaboration with local EMS services. Printed on durable yellow paper in a standardized credit-card format to ensure high visibility, it allows patients to select one of six predefined categories representing different levels of desired intervention, ranging from full intensive care (category 1) to DNR/ DNI order with treatments such as anti-infective therapy in the outpatient setting, e.g. at home (category 5), to exclusive palliative measures with no further treatments except for symptom control at home (category 6). By focusing on essential information, the card benefits both patients—by ensuring that their wishes are honored—and emergency physicians—by improving clarity and confidence in time-critical decision-making.

The growing demand for specialized care focused on symptom control and quality of life due to the aforementioned demographic and epidemiological shifts is reflected in a growing utilization of palliative care services in Germany [5]. Palliative care is dedicated to treating patients with incurable, progressive, and advanced diseases with limited life expectancy. Its holistic approach aims to improve the patient’s and next-of-kin’s quality of life [6, 7] and addresses not only physical symptoms but also the psychological, social, and spiritual dimensions of suffering [8].

In Germany, palliative care is provided either as inpatient care in one of the 340 palliative care units nationwide [9], or at home through Specialized Outpatient Palliative Care (SOPC). All individuals covered by statutory health insurance are entitled to SOPC (Section 37b of the German Social Code V), and it is estimated that up to 10% of all palliative patients receive SOPC due to severe symptoms affecting their quality of life [10, 11].

SOPC teams provide specialized medical and nursing care with 24/7 availability to manage complex symptoms. This includes medication management, psychosocial support (where available), assistance for next of kin, and the coordination of other healthcare providers involved in patient care. Furthermore, they assist patients with the development of emergency plans and with preparing advance directives, thereby preventing inappropriate or unwanted hospital admissions and over-treatment [12, 13] and enabling patients to die in their preferred home setting [12]. Besides these positive effects on patient well-being, this also reduces strain on EMS and EDs.

The aim of this study is to analyze the impact of SOPC on the completion of advance care documents, including living will, lasting powers of attorney, and medical emergency ID cards. Additionally, the study seeks to assess the influence of documented patient preferences on hospital admissions.

## Methods

### Ethical approval and study population

The Ethics Committee of the Medical Faculty at Heinrich Heine University Düsseldorf granted ethical approval for this study on October 4, 2023 (Study No.: 2023–2490). All adult patients who were referred to the SOPC service at the University Hospital Düsseldorf between July 1, 2022 and June 30, 2023 were included in this retrospective cohort study.

### Data collection

Data collection was performed using the “PalliDoc” documentation system (StatConsult Gesellschaft für klinische und Versorgungsforschung mbH, Magdeburg, Germany, Version 1.8.3f), which is utilized by the SOPC team at University Hospital Düsseldorf, Germany. Demographic data, “Pflegegrad” (*level of care dependency* – a scoring system used in Germany ranging from 1 to 5, with 5 indicating the highest level of professional care required), information on advance care directives, hospitalizations, adherence to patient wishes (no radiation, no chemo- or immunotherapies, no operations), treatments, and symptoms were extracted from the digital records of the included patients and entered into a pseudonymized table using Excel (Microsoft Corporation, 2024). Upon completion of data analysis, the key linking patient identities to the pseudonymized data was destroyed.

### Data analysis

Descriptive and inferential statistical analyses were performed using JASP statistical software (Version 0.18.3). For nominal and ordinal variables, absolute and relative frequencies were calculated. The metric variables were analyzed using the arithmetic mean, median, minimum (Min.), maximum (Max.), interquartile range (IQR), and normality tested using the Shapiro-Wilk test. Skewness was calculated to describe the distribution of metric variables. For the metric variable of hospital admissions, a frequency distribution was created to determine how many patients were hospitalized once or multiple times. A significance level of *p* < 0.05 was considered statistically significant for all tests. Differences between patient groups were examined using the Chi-square test and the Mann-Whitney U test. Effect sizes were calculated using Cramer’s V and Pearson’s correlation coefficient. The question of whether the medical emergency ID card was created significantly more often during SOPC was addressed using a binomial test.

## Results

### Demographics

359 patients were included in the study. The demographic data are summarized in Table 1. The average age at the start of SOPC was 73.6 years (SD: 13.4, median: 75 years, IQR: 17 years, minimum: 27 years, maximum: 100 years). The age distribution of the patients did not follow a normal distribution. A small proportion of patients (3.3%, *n* = 12) were under SOPC for only one day, and three patients (0.8%) died on the day of admission. These values were also not normally distributed. Further data are summarized in Table 1.

**Table 1:** Demographic data of included patients

	Characteristics	*n* (%)
**sex**	Female	187 (52.0%)
	Male	172 (48.0%)
**Age [years]**	20–29	4 (1.1%)
	30–39	5 (1.4%)
	40–49	8 (2.2%)
	50–59	32 (8.9%)
	60–69	71 (19.8%)
	70–79	103 (28.7%)
	80–89	103 (28.7%)
	90+	33 (9.2%)
**Time in SOPC [days]**	Average duration	41.6
	Median	23
	SD	51.4
	IQR	43
	Q1	9
	Q2	23
	Min.	1
	Max.	362
**Living situation**	Alone	79 (22.6%)
	With spouse	131 (37.4%)
	With partner	13 (3.7%)
	With family	24 (6.9%)
	Nursing home	53 (15.1%)
	Hospice	50 (14.3%)
	*Data unavailable*	9 (2.5%)
**Social status**	Single	163 (45.9%)
	Married	176 (49.6%)
	Civil partner	3 (0.8%)
	Partner	13 (3.7%)
	*Data unavailable*	4 (1.1%)

### Diseases, treatments and levels of care dependency

The predominant disease category among the patients was cancer, with 280 patients (78.0%) affected. During the observation period, 61 patients (17.0%) received at least one radiation, chemotherapy, or immunotherapy session. The most common form of treatment was chemotherapy (10.9%, *n* = 39/359), followed by radiation therapy (6.7%, *n* = 24/359) and immunotherapy (4.5%, *n* = 16/359). For 291 patients, the level of care dependency was documented: most of them were classified as having care level 3 (*n* = 122, 41.9%). Table 2 provides further details on disease categories and care levels, Table 3 shows the distribution of care levels among disease entities.

**Table 2:** Diseases and levels of care dependency (others: lupus erythematosus and Fournier gangrene)

	Characteristics	*n* (%)
Disease Entity (*n* = 359)	Cancer	280 (78.0%)
	Neurological	30 (8.4%)
	Cardiovascular	29 (8.1%)
	Pulmonary	14 (3.9%)
	Gastrointestinal	4 (1.1%)
	Others	2 (0.6%)
„Pflegegrad“; level of care dependency (*n* = 291)	Level 1	7 (2.4%)
	Level 2	63 (21.6%)
	Level 3	122 (41.9%)
	Level 4	67 (23.0%)
	Level 5	32 (11.0%)

**Table 3:** Levels of care among disease entities (other: lupus erythematosus and Fournier gangrene)

Disease entity	Level of care 1 *n* (%)	Level of care 2 *n* (%)	Level of care 3 *n* (%)	Level of care 4 *n* (%)	Level of care 5 *n* (%)	Missing *n*
**Cancer**	7 (3.2)	50 (22.5)	98 (44.1)	50 (22.5)	17 (7.7)	58
**Neurological**	0 (0.0)	2 (7.7)	8 (30.8)	5 (19.2)	11 (42.3)	4
**Cardiovascular**	0 (0.0)	8 (32.0)	7 (28.0)	7 (28.0)	3 (12.0)	4
**Pulmonary**	0 (0.0)	2 (15.4)	7 (53.8)	3 (23.1)	1 (7.7)	1
**Gastrointestinal**	0 (0.0)	0 (0.0)	1 (33.3)	2 (66.7)	0 (0.0)	1
**Others**	0 (0.0)	1 (50.0)	1 (50.0)	0 (0.0)	0 (0.0)	0

### Advance directives

Among the 359 patients, 117 (32.6%) had a medical emergency ID card during the observation period. Patients aged 70 to 89 years had the highest prevalence of completed medical emergency ID cards. For 12 cases, the date of setting up the ID card was retrospectively not assessable. Out of the remaining 105 analyzed documents, 21 (20.0%) were completed before the initiation of SOPC and 84 (80.0%) while receiving SOPC. The medical emergency ID card was thus significantly more likely to be created during SOPC than prior to SOPC involvement (*p* < 0.05). Table 4 shows the categories on the medical emergency ID and the distribution of their selection by patients. The most frequently chosen category was category 6 (*n* = 43/114, 37.7%). In general, older patients were attributed higher categories for the medical emergency ID card (*p* = 0.003) (see Table 5). No patient changed the category in their emergency ID card during the observation period. Due to incomplete documentation, the category of three ID cards was not retrievable.

**Table 4:** Categories selected on the medical emergency ID card (*n* = 114)

Category	Desired Measures	*n* (%)
1	Maximum Emergency and Intensive Care Transfer to Hospital	5 (4.4%)
2	Do Not Resuscitate (DNR) Transfer to Hospital	4 (3.5%)
3	Do Not Resuscitate (DNR) Do Not Intubate (DNI) Transfer to Hospital	10 (8.8%)
4	Do Not Resuscitate (DNR) Do Not Intubate (DNI) No Treatment in the Intensive Care Unit Transfer to Hospital	30 (26.3%)
5	Do Not Resuscitate (DNR) Do Not Intubate (DNI) No Treatment in the Intensive Care Unit No Transfer to Hospital (outpatient therapy)	22 (19.3%)
6	Exclusively palliative (comfort) measures (no oxygen) No transfer to hospital	43 (37.7%)

**Table 5:** Emergency ID card – Age and predominant category

Age [years]	Prevalence [*n*; %]	Emergency ID category 1–3 [*n*; %]	Emergency ID category 4–6 [*n*; %]
30–39	1; 0.9	0; 0	1; 100
50–59	13; 11.1	3; 23.1	10; 76.9
60–69	20; 17.1	6; 30	14; 70
70–79	32; 27.4	6; 18.8	26; 81.2
80–89	35; 29.9	6; 17.1	29; 82.9
> 90	16; 13.7	0; 0	16; 100
**Total**	**117; 100**	**21; 17.9**	**96; 82.1**

By the end of the observation period, 74.9% (*n* = 269) of the 359 patients had a documented lasting power of attorney. Most of these (89.2%, *n* = 240) had already been created before starting SOPC. A living will was documented for 46.8% (*n* = 168) of all 359 patients by the end of the observation period. Almost all of these (95.5%, *n* = 160) had been completed before the onset of SOPC.

### Hospital admissions

During the observation period, 79.7% (*n* = 286) of patients were not admitted to the hospital. Of those who were hospitalized, 14.8% (*n* = 53) had a single hospital admission, while 5.5% (*n* = 20) experienced multiple hospitalizations. Those patients without hospitalizations were older than those with one or more hospital admissions (*p* < 0.05, *r* = 0.154). Patients with oncological diseases were more likely to be hospitalized than those with non-oncological conditions (*p* < 0.05, Cramer’s V = 0.190). There was a correlation between the selected category in the Emergency ID card and the number of hospital admissions (*p* < 0.05; *r* = 0,376). Patients who were classified as category 5 or 6 in the medical emergency ID card after weighing up the indication and patients’ wishes were significantly less likely to be hospitalized than those who had documented categories 1 to 4 (*p* < 0.05, *r* = 0.376).

### Place of death

The place of death was retrospectively retrievable for *n* = 263 patients. Most of them died either in their own home (*n* = 99/263, 37.6%) or in a hospice (*n* = 81/263, 30.8%). A comparatively small proportion of patients died in nursing homes (*n* = 39/263, 14.8%). The hospital was the place of death in 16.7% (*n* = 44/263) of all cases, and more patients died on the palliative care ward (*n* = 24/263, 9.1%) than on other wards (*n* = 20/263, 7.6%).

## Discussion

In this retrospective longitudinal study, 359 patients who were cared for by the SOPC team at the University Hospital Düsseldorf from July 1, 2022 to June 30, 2023 were analyzed regarding the establishment of advance directives and their impact on the adherence to patients’ wishes concerning hospitalizations.

The previously described dominant condition in SOPC is cancer, while the most common non-oncological conditions are heart failure, followed by chronic obstructive pulmonary disease (COPD), renal failure, and dementia. Similarly, in this cohort, cancer accounted for 78.0% of cases. The average age of 73.6 (SD 13.4) years is consistent with other cohorts, where the average age is typically over 70 years, making our cohort comparable to others [14].

In this cohort, 46.8% of patients had a living will, and 74.9% had a lasting power of attorney. During the study period, only 32.6% (*n* = 117) of all patients had a medical emergency ID card with only 5.8% (*n* = 21) having it completed before the initiation of SOPC. This aspect prompted a revision of standard operating procedures within the observed SOPC team: Medical emergency ID cards are now systematically discussed with newly admitted patients, likely resulting in a higher prevalence of completed Emergency ID cards in the future.

Barriers to the completion of advance directives by patients have already been investigated in scientific studies. Some of these have been explored by Breen et al. [15] as part of their prospective observational study on the presence of living wills and powers of attorney, which included focus group interviews with physicians and nurses from a palliative care service. The study identified a general lack of awareness regarding the benefits of advance directives. In addition, there was misunderstanding about the fact that an advance directive is valid even without notarization. Another barrier was the fear that treatment could be unnecessarily limited. Patients also expressed concern about the potential for disempowerment despite retaining decision-making capacity, and for decisions to be made against their wishes [15]. Further research is needed to explore the reasons for this. However, our data suggest that SOPC teams might be helpful in breaking down these barriers as they facilitate the completion of advance directives, in our cohort primarily medical emergency ID cards.

Advance care planning focuses on goal-concordant care at the end of life for patients. It is a process, meaning that patients have several talks with trained professionals in order to understand and share one’s values and preferences. Making medical decisions and setting up advance directives should be discussed thoroughly and the process should be well-documented [16, 17].

Emergency physicians seem to see benefits in medical emergency cards, and when surveyed supported a wider introduction [18]. Unfortunately, to date their use is only established in a handful of German regions [19], and while similar emergency advance directives have been implemented elsewhere in Germany, scientific studies on the topic remain scarce [18]. In another survey of 383 emergency physicians, only 16.2% answered hat standardized emergency advance directives are available in the catchment area of their emergency department [20]. In the future, efforts should be made to connect patients and their care-takers as well as health-care providers to develop a universally established and accepted medical emergency ID. Additionally, public health campaigns should raise awareness about the availability of such emergency cards among the general public and actively encourage their utilization.

International studies have shown that SOPC helps reduce unnecessary hospital admissions and treatments [21, 22]. Due to a lower rate of utilization of inpatient healthcare services, the integration of SOPC services leads to a more cost-efficient attribution of resources [23]. However, German studies on the cost-effectiveness of SOPC compared to inpatient care are not yet available. Nonetheless, our study demonstrates a low hospitalization rate and adherence to patients’ wishes, as documented in medical emergency ID cards. This confirms the patient- and needs-oriented care provided by SOPC.

In the general population, hospitals represent the most common place of death, accounting for over 50% of cases, while the home setting is the second most common at only 21.7% [24]. In contrast, patients in SOPC are significantly more likely to die at home [7, 12]. Depending on how the home setting is defined across studies, this is achieved in approximately two-thirds of cases [12, 25, 26], and in a large analysis of 14,460 patients from 14 SOPC teams, 85.9% died at home and onl 7.7% of patients died in the hospital [12]. This is consistent with our data, as only 16.7% of the patients in our cohort died in a hospital.

### Limitations

This study is based on a retrospective design, and therefore, the primary and secondary endpoints were adapted to available data. Due to partially insufficient documentation, not all data for the medical emergency ID cards or place of death could be collected retrospectively. It was not possible to further investigate why some patients did not create a medical emergency ID card or why patients who excluded hospital admission in their medical emergency ID card were hospitalized after all. Future research should include qualitative data to gain a nuanced understanding of barriers to the creation of advance care directives, especially medical emergency ID cards. Furthermore, the analysis is based on data from a single center, so the results provide only a trend for Germany, and future studies should involve a broader data set.

## Conclusion

Specialized Outpatient Palliative Care (SOPC) plays a key role in supporting patients with life-limiting illnesses, many of whom experience complex and difficult-to-manage symptoms. By providing care at home SOPC helps to prevent overtreatment at the end of life.

Advance care directives further support this goal by ensuring that medical interventions align with patient preferences. The medical emergency ID card is particularly valuable in this context: it is easy to carry and provides a clear, readily available representation of the patient’s wishes. In our study, patients with an exclusion of hospital admission in their medical emergency ID card showed significantly reduced hospitalization rates. Importantly, the card also provides other healthcare providers—such as EMS personnel and emergency physicians—with clarity and legal certainty in time-critical situations. In our study, the majority of these cards were completed during the course of SOPC, highlighting the proactive role of palliative care teams in preparing for emergencies. There is a need for greater awareness among health care professionals of the benefits and availability of such emergency ID cards so that all patients with life-limiting illness can benefit from them in the future. Broader implementation would not only improve patient-centered emergency care but also reduce the burden on EMS and emergency departments, contributing to more efficient use of healthcare resources.

## Data Availability

No datasets were generated or analysed during the current study.

## Abbreviations

DNI: Do Not Intubate
DNR: Do Not Resuscitate
ED: Emergency department
EMS: Emergency medicine services
ID: Card Identification card
IQR: Interquartile range
Max.: Maximum
Min.: Minimum
SOPC: Specialized outpatient palliative care
